# Symptom clusters, associated factors and health‐related quality of life in patients with chronic obstructive pulmonary disease: A structural equation modelling analysis

**DOI:** 10.1111/jocn.16234

**Published:** 2022-01-30

**Authors:** Fei Fei, Richard J Siegert, Xiaohan Zhang, Wei Gao, Jonathan Koffman

**Affiliations:** ^1^ Florence Nightingale Faculty of Nursing Midwifery and Palliative Care Cicely Saunders Institute King’s College London London UK; ^2^ 605090 School of Nursing and Midwifery Jiangsu College of Nursing Huai'an Jiangsu China; ^3^ Faculty of Health and Environmental Sciences Auckland University of Technology Auckland New Zealand

**Keywords:** chronic obstructive pulmonary disease, health‐related quality of life, structural equation modelling, symptom assessment, symptom cluster

## Abstract

**Aims and objectives:**

To identify symptom clusters and develop a symptom cluster model among people living with chronic obstructive pulmonary disease (COPD).

**Background:**

The examination of symptom clusters in COPD patients is an emerging field of scientific inquiry directed towards symptom management. However, no studies have modelled the relationships among symptom clusters, associated factors and health‐related quality of life.

**Design:**

A cross‐sectional design with convenience sampling following STROBE guidelines.

**Methods:**

Data were collected from 450 COPD participants in three university teaching hospitals. Participants were invited to complete a structured questionnaire comprised of a socio‐demographic/clinical questionnaire, Integrated Palliative Care Outcome Scale and Clinical Respiratory Questionnaire. Exploratory factor analysis and confirmatory factor analysis were used to identify symptom clusters. Structural equation modelling was used to examine the proposed model.

**Results:**

The respiratory related symptom cluster, psychological symptom cluster and cough‐insomnia related symptom cluster were identified. The final model demonstrated a good fit with the data. Gender, stage of disease and monthly income were significant factors associated with symptom clusters. Respiratory related and cough‐insomnia related symptom clusters had a direct negative impact on health‐related quality of life, while the psychological symptom cluster was found to have a direct and indirect negative effect on health‐related quality of life.

**Conclusions:**

Final COPD symptom cluster model should serve as a framework to guide intervention research targeting symptom clusters to improve health‐related quality of life of people living with COPD.

**Relevance to Clinical Practice:**

Nurses should be especially attuned to identify those at most risk of facing a higher symptom burden in this case those who are female, have advanced stage COPD and/or lower income. During the clinical symptom assessment, nurses should pay attention to the close relationships among symptoms within a cluster to identify any ‘trigger’ symptom that could cause the development or exacerbation of other symptoms.

**What does this paper contribute to the wider global clinical community?**

- Three symptom clusters have been identified in COPD patients: respiratory related symptom cluster (shortness of breath, weakness, drowsiness and poor mobility); psychological symptom cluster (anxiety and depression); and cough‐insomnia related symptom cluster (cough, dry mouth, poor appetite and insomnia).
- This study provides new evidence that in order to identify those at most risk of facing a higher symptom burden leading to poor quality of life, nurses should be especially attuned to consider those patients with COPD who are female, have an advanced stage COPD and/or low‐income.
- This study is the first to develop a symptom cluster model in COPD patients serving as a framework to progress and guide new lines of assessment, appropriate interventions and research to improve health‐related quality of life.

## 1 INTRODUCTION

Chronic obstructive pulmonary disease (COPD) is a major chronic disease, highly prevalent in ageing populations exposed to tobacco smoke and airborne pollutants (Vos et al., 2012). The 2022 Global Initiative for Chronic Obstructive Lung Disease (GOLD) strategy document defines COPD as ‘*a common*, *preventable and treatable disease that is characterized by persistent respiratory symptoms and airflow limitation that is due to airway and*/*or alveolar abnormalities usually caused by significant exposure to noxious particles or gases*.*’* (Global Initiative for Chronic Obstructive Lung Disease, 2022). The Global Burden of Disease (GBD) Study 2015 estimated that the global prevalence of COPD was approximately 174 million cases and that 3.2 million people died from COPD worldwide (Naghavi et al., 2017). It has been projected that by 2030 COPD will become the third‐highest cause of death (Quaderi & Hurst, 2018). In addition, Global Initiative for Chronic Obstructive Lung Diseases (GOLD) 2022 reported, globally, the prevalence of COPD is expected to rise over the next 40 years and by 2060 there may be 5.4 million deaths annually from COPD (Global Initiative for Chronic Obstructive Lung Disease, 2022).

Published studies from high‐income countries have reported large variations in the prevalence and incidence of COPD, both within countries and also among different countries. For example, data from the USA estimate prevalence to vary from 10.2%–20.9% (Fan et al., 2017; Tilert et al., 2013). In Europe, when adhering to the GOLD criteria, the prevalence of COPD has been shown to range widely from 3% in the Netherlands (Afonso et al., 2011) to 26.1% in Austria (Schirnhofer et al., 2007). COPD has generally been poorly studied in Africa, particularly in sub‐Saharan countries. However, where research has been conducted prevalence has been shown to vary from 1.6% in Cameroon (Mbatchou Ngahane et al., 2015) to 23.8% in South Africa (Nkosi et al., 2015). There is a lack of data from the Middle East and South Asia on under‐ and over‐diagnosis of COPD, similar to that from the Africa region (Ho et al., 2019). The prevalence of smoking in Central and South America is high, with a median prevalence of 30%, contributing significantly to the burden of COPD where the prevalence is as high as 31.1% in Brazil (Menezes et al., 2004; Soriano et al., 2017). With the exception of China, where it has been estimated the prevalence of COPD is 8.6%, corresponding to 99.9 million COPD patients aged 20 years or older (Wang et al., 2018), research of the prevalence of COPD in many countries in south‐east Asia is still quite limited. Specifically, COPD prevalence estimates vary widely in Asian countries, with 3.5% in Hong Kong and Singapore, 5% in Thailand, 5.4% in Taiwan, 5.6% in Indonesia, 5.9% in South Korea and 6.1% in Japan, 6.3% in the Philippines and 6.7% in Vietnam (Regional COPD Working Group, 2003). Health‐related quality of life (HRQoL) refers to people’s perceptions of their physical, psychological, and social function and well‐being (World Health Organization Quality of Life Group [WHOQOL Group], 1995). Previous studies have shown that the substantial symptom burden of COPD reduces the mental health and physical function of people living with COPD, ultimately lowering their HRQoL which is considered to be an important outcome of treatment and care (Henoch et al., 2016).

## 2 BACKGROUND

People with COPD experience multiple distressing symptoms including breathlessness, fatigue, anorexia, pain, cough, poor mobility, insomnia, dry mouth, among others (Maddocks et al., 2017). A United States, population‐based, longitudinal study of 98 adults with COPD found that respiratory symptoms such as cough and breathlessness may be responsible for poor sleep quality (Omachi et al., 2012); an observational, cross‐sectional, multicentre study investigating factors associated with depression and anxiety in Spain COPD found that patients with depression had greater dyspnoea than those without (Miravitlles et al., 2014). Moreover, another 3‐year multicentre study, that investigated 2118 subjects with COPD in 46 centres from 12 countries including the United States, United Kingdom, Denmark, Netherlands among others, aimed to identify the determinants of depression and identified the significant association between fatigue and depression (Hanania et al., 2011). Therefore, symptom management for people living with COPD is especially challenging since they often experience multivariate symptom relationships (Eckerblad et al., 2014). This phenomenon is referred to as a ‘symptom cluster’ and implies a stable group of two or more interrelated symptoms occurring simultaneously, which may or may not share the same aetiology (Kim et al., 2005). Emerging evidence in COPD suggests that different symptoms might be alleviated by the same strategy. For example, the pulmonary rehabilitation approach is used for alleviating breathlessness and fatigue (Baltzan et al., 2011); and cognitive behavioural techniques can be used for alleviating anxiety and depression (Chochinov et al., 2011). Therefore, the identification of symptom clusters has become an emerging and important field of scientific inquiry (Dong et al., 2014). For people with COPD and the nurses who care for them, the desire for optimal assessment and management has driven researchers to identify symptom clusters associated with this disease (Kwekkeboom, 2016). This should lead to more favourable person‐centred experiences and outcomes of care (Miaskowski et al., 2017). Our recent systematic review identified multiple research designs, measurement tools and statistical methods, employed to investigate this phenomenon, which supports this view. However, it also highlighted inconsistencies and a lack of precision in the actual composition of the symptom clusters identified (Fei et al., 2021). Furthermore, previous studies have only made use of a narrow spectrum of potential symptoms present in COPD (Lim et al., 2017; Park & Larson, 2014; Wu et al., 2020; Yang et al., 2020), which limits our understanding of this issue. In particular, they have overlooked certain symptoms, including poor mobility, which is highly prevalent among people with COPD (Shay et al., 2020). Moreover, the sample size of the original research studies designed to investigate and identify the COPD symptom clusters ranged from 54 to 250 (Fei et al., 2021). This could be regarded as being inadequate to produce reliable and generalisable results. Therefore, some researchers have suggested that the use of combined or big data analysis may represent one solution to mitigate this concern (Bakken & Reame, 2016). This should be accompanied by greater attention to more sophisticated data analysis techniques (Corwin et al., 2014).

In the present study, we adopted the Theory of Unpleasant Symptoms (TOUS) as the guiding conceptual framework. The TOUS is comprised of three overarching concepts: (i) influencing factors, (ii) symptoms and (iii) performance (Lenz et al., 1997). Specifically, the TOUS assumes that *symptoms* can occur alone or with multiple other symptoms frequently resulting in multiple relationships between and among symptoms (Lenz & Pugh, 2014). *Influencing factors* are those physiological, psychological and situational factors that influence the symptoms; the concept of *performance* is illustrated as the effects or outcomes of the symptom experience, including functional and cognitive activities (Lenz, 2018). According to the TOUS, it includes ‘feedback loops’ to indicate that influencing factors can affect the symptom experience, symptoms can have consequences and consequences can, in turn, affect influencing factors and the symptom experience (Lenz & Pugh, 2014; Lenz et al., 1997). In addition, some of the critiques of the TOUS have indicated that failure to include HRQoL as an explicit outcome is an inherent weakness (Brant et al., 2010). Research has identified that symptoms influence not only the physical but also the affective aspects of quality of life and that mood may both impact and be impacted by symptoms (Blakeman, 2019). Therefore, researchers suggest the need to consider adding affective outcomes and/or HRQoL to the TOUS in symptom research (Brant et al., 2010). Moreover, what we do know is the presence of symptom clusters among people living with COPD has a significant and deleterious impact on HRQoL (Wu et al., 2020). This links to the challenge many nurses experience when attempting to alleviate the symptom burden of COPD, possibly due to their inadequate understanding of the relationships between symptom clusters and associated factors (e.g. age, stage of disease and income; Fei et al., 2021). Characterising the relationships among these factors and symptom clusters is of great importance in guiding the development of precise and effective interventions to improve the well‐being of people with COPD.

This paper, therefore, aimed to examine and investigate the relationships among symptom clusters, associated factors and HRQoL in COPD patients, while addressing the following specific questions: (i) what is the composition of symptom clusters in COPD patients? (ii) what represents the best model to illustrate the interaction among these symptom clusters, relevant associated factors and HRQoL in COPD patients?

## 3 METHODS

### 3.1 Design and participants

**FIGURE 1:**
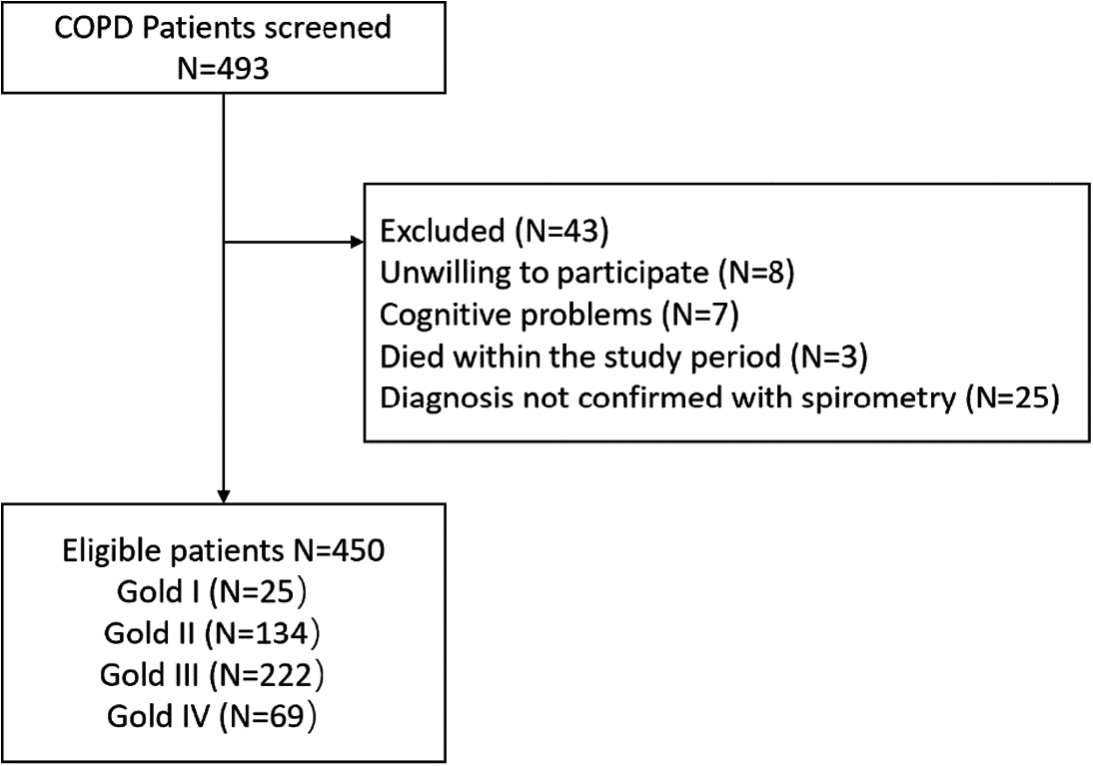
Flowchart of the cross‐sectional study

This study represents a multicentre, cross‐sectional survey using convenience sampling to develop a symptom cluster model in COPD patients based on the TOUS following the Strengthening the Reporting of Observational Studies in Epidemiology (STROBE) guidelines (Supplementary File 1; Von Elm et al., 2014). This study was conducted in three tertiary‐level university teaching hospitals in Jiangsu Province of Eastern China. According to the latest survey report published in 2021, the prevalence of COPD in people 40 years of age or older in Jiangsu is 11.9% (Su et al., 2021) that closely corresponds with the overall prevalence of COPD (8.6%) in China (Wang et al., 2018). Eligibility criteria included the following: (i) a primary diagnosis of COPD given by clinicians according to the post‐bronchodilator spirometry in pulmonary function test and forced expiratory volume in one second (FEV1)/ Forced vital capacity (FVC) ≤70%; (ii) potential participants must be 18 years or older and (iii) the ability to provide written informed consent. Initially, 493 people with COPD were recruited between October 2019 and May 2020, of whom 43 were excluded (see Figure 1). Post hoc power analysis using G‐Power 3.1.7 indicated that 450 study participants yielded 100% power with an effect size (f**^2^**) of 0.85, calculated by a formula using multiple R**^2^** at a significance level of 0.05 (two‐sided; Verma & Verma, 2020).

### 3.2 Ethical considerations and data collection

We obtained ethical approval for our study from King's College London (approval number: HR‐18/19‐13608). We also obtained ethical approvals from the Second People's Hospital of Changzhou Ethics Committee (approval number: [2019] KY063‐01), the Affiliated Huai'an No.1 People's Hospital of Nanjing Medical University Ethics Committee (KY‐P‐2019–048–01) and the Affiliated Huai'an Hospital of Xuzhou Medical University Ethics Committee (approval number: HEYLL201932).

All selected eligible people with COPD were informed about the purpose of the study, method of data collection used, their potential time commitment, and any possible benefits and risks associated with the study. For those who chose to participate in the study, trained data collectors obtained their written informed consent and subsequently conducted face‐to‐face interviews. The presence of missing data was mitigated by providing detailed training for the data collectors in the study documentation, double‐checking collected data and prompt follow‐up phone calls with study participants where missing data were identified.

### 3.3 Measures

A socio‐demographic and clinical questionnaire was used for assessing the physiological and situational characteristics of participants, particularly, gender, age, education level, income, number of comorbidities and stage of COPD. Our systematic review indicated that there was no ‘gold standard’ assessment tool to evaluate symptoms in COPD patients (Fei et al., 2021).

The Integrated Palliative care Outcome Scale (IPOS, patient version) includes 17 items covering physical symptoms (pain, shortness of breath, weakness, nausea, vomiting, poor appetite, constipation, dry mouth, drowsiness and poor mobility), psychological symptoms (anxiety, depression), feeling at peace, family distress, sharing feelings with family or friends, information needs and practical issue (Murtagh et al., 2019). Developed from integrating the original Palliative Outcomes Scale (POS), the tool has been validated for use in multiple types of serious and/or chronic illnesses and is widely used in palliative care (Jeffery, 2018). In this study, we combined physical symptoms and psychological symptoms as a comprehensive symptom list. Of note, according to the literature review (Fei et al., 2021; Maddocks et al., 2017) and the researchers’ clinical experience, we added another two common physical symptoms (cough and insomnia) to the symptom list, and subsequently, 14 IPOS items (a list of 14 symptoms) containing 12 physical symptoms and 2 psychological symptoms used to assess symptoms. In this study, Cronbach's alpha for the 14 IPOS items was 0.77, which indicated acceptable internal consistency.

HRQoL was assessed using the ‘Clinical Respiratory Questionnaire’, which is a disease‐specific HRQoL questionnaire focusing on measuring respiratory health status (Reda et al., 2010). Content validity of the CRQ has been supported by the opinions from respiratory experts (Chan et al., 2006); and the convergent and discriminant validity of the CRQ were supported by the correlations between individual items and their corresponding domain scores, as well as the much lower correlations between the items and scores of non‐corresponding domains (Meng et al., 2011). In a previous study, the Cronbach's alpha values for the four dimensions of the CRQ ranged from 0.75 to 0.84 (Chan et al., 2006). In this study, its Cronbach's alphas were 0.90, 0.86, 0.72 and 0.87 for the dyspnoea, fatigue, mastery and emotional function domains of the CRQ.

### 3.4 Data analysis

Descriptive statistics, specifically means, standard deviations, frequency and percentages were calculated for the physiological characteristics, situational characteristics and symptom experience profile. Symptoms with prevalence >20% were selected from 14 IPOS items for exploratory factor analysis (EFA) for the identification of symptom clusters (Murtagh et al., 2019). For EFA, Varimax rotation was used to extract the components from a principal component analysis after first verifying the factorability of the data via the Kaiser–Myer–Olkin (KMO) test (value > 0.60) and the significance of Bartlett's Test of Sphericity (Hair et al., 2014). The number of factors (clusters) was determined by examining factors with eigenvalues >1.0, using 0.50 for factor loadings as the minimum cut‐off value, to enhance the interpretation of clustering (Hair et al., 2014). Confirmatory factor analysis (CFA) was used to test the goodness‐of‐fit of the EFA results (Orcan, 2018).

Finally, structural equation modelling was undertaken to examine the potential relationships among symptom clusters, associated factors and HRQoL. The fit indices used to evaluate the proposed model were normed chi‐square divided by degrees of freedom (χ^2^/df), the Comparative Fit Index (CFI), the Tucker–Lewis Index (TLI), the Root Mean Square Error of Approximation (RMSEA) and finally the Standardised Root Mean Square Residual (SRMR; Kline, 2015). The model was considered to have an adequate fit when the χ^2^/df was <5, the value of the CFI and TLI were >0.90 and both RMSEA and SRMR values were <0.08 (Kline, 2015). *p*‐value <.05 was considered statistically significant. STATA/IC 15.1 was used for all statistical analyses.

## 4 RESULTS

### 4.1 Sample characteristics and symptom profile

In total, 450 participants completed the study questionnaire. Of these, 321 (71.3%) were male, and the average age was 73.7 years (SD = 9.2; range: 33–95 years). The characteristics of study participants are presented in Table 1 and the prevalence of COPD‐related symptoms and associated distress is presented in Table 2.

**TABLE 1:** Socio‐demographic and clinical characteristics of the study sample (*N* = 450)

Characteristics	Patients	
	*n*	%
Sex, *n* (%)		
Male	321	71.3
Female	129	28.7
Age in years, *n* (%)	(Mean 73.7; median 75)	(SD 9.2; range 33–95)
<60	29	6.4
60–70	128	28.4
71–80	183	40.8
>80	110	24.4
Marital status, *n* (%)		
Not married	13	2.9
Married	380	84.4
Divorced or widowed	57	12.7
Educational level, *n* (%)		
Uneducated	95	21.1
Elementary school	150	33.3
Middle/high school	166	36.9
Vocational school	23	5.1
≥College	16	3.6
Living state, *n* (%)		
Alone	56	12.4
Living with spouse	246	54.8
Living with other family members	144	32.0
Living in private welfare institutions	2	0.4
Living in social welfare institutions	2	0.4
Hospitalisation cost, *n* (%)		
Publicly funded free medical care	24	5.3
Medical insurance	411	91.3
Commercial insurance	3	0.7
Self‐funded	12	2.7
Monthly income per person (Chinese Yuan), *n* (%)		
<500	163	36.2
500–1000	37	8.2
1000–3000	110	24.5
>3000	140	31.1
Employment state		
Employed	12	2.7
Temporarily employed/on sick leave	1	0.2
Unemployed	29	6.4
Farming	157	34.9
Retired	251	55.8
Time since diagnosed (Years), *n* (%)	(Mean 14.4; median 10)	(SD 10.9; range 1–60)
1–10	254	56.4
11–20	117	26.0
21–30	44	9.8
>30	35	7.8
Home oxygen therapy		
Equipped	217	48.2
Unequipped	233	51.8
Smoking history, *n* (%)		
Current smoker	68	15.1
Never smoker	154	34.2
Former smoker (quit smoking)	228	50.7
Alcohol history, *n* (%)		
Current drinking	52	11.6
Never drinking	231	51.3
Former drinking	167	37.1
Number of comorbidities, *n* (%)		
None	69	15.3
One	105	23.3
Two	108	24.0
≥Three	168	37.3
FEV1 in % predicted, *n* (%)		
Gold I (Mild)	25	5.6
Gold II (Moderate)	134	29.8
Gold III (Severe)	222	49.3
Gold IV (Very severe)	69	15.3

**TABLE 2:** Prevalence and associated distress of symptoms in COPD patients (*N* = 450)

Symptoms		Frequency (%)					Mean (SD)	Median
	Prevalence*	Not at all (0)	Slightly (1)	Moderately (2)	Severely (3)	Overwhelmingly (4)		
Pain	18.7	57.7	23.6	10.8	7.2	0.7	0.70 (0.97)	0
Shortness of breath	89.5	0.9	9.6	22.0	45.5	22.0	2.78 (0.93)	3
Weakness	80.4	3.6	16.0	24.0	45.1	11.3	2.45 (1.01)	3
Nausea	10.2	80.0	9.8	7.8	2.0	0.4	0.33 (0.74)	0
Vomiting	6.2	84.0	8.9	5.3	1.6	0.2	0.25 (0.65)	0
Poor appetite	35.7	38.7	25.6	17.6	15.8	2.3	1.18 (1.18)	1
Constipation	16.6	65.6	17.8	10.2	5.3	1.1	0.59 (0.95)	0
Sore or dry mouth	59.9	19.3	20.8	28.4	25.1	6.4	1.79 (1.20)	2
Drowsiness	46.5	24.4	29.1	21.6	20.9	4.0	1.51 (1.18)	1
Poor mobility	70.0	9.8	20.2	26.0	27.1	16.9	2.21 (1.22)	2
Cough	66.5	7.3	26.2	28.2	29.3	9.0	2.06 (1.10)	2
Insomnia	44.2	32.9	22.9	18.9	20.2	5.1	1.42 (1.27)	1
Anxiety	53.3	26.7	20.0	35.8	14.9	2.6	1.47 (1.11)	2
Depression	28.5	40.4	31.1	22.7	4.7	1.1	0.95 (0.96)	1

Abbreviation: SD, standard deviation

* Prevalence was defined as any IPOS symptoms specified as ‘moderate’, ‘severe’ or ‘overwhelming’.

### 4.2 Symptom clusters

#### 4.2.1 Exploratory factor analysis (EFA)

According to the prevalence of symptoms displayed in Table 2, shortness of breath, weakness, poor appetite, dry mouth, drowsiness, poor mobility, cough, insomnia, anxiety and depression were selected for EFA. We observed that Bartlett's test of sphericity was significant (*p *< .05), and the Kaiser–Meyer–Olkin (KMO) was 0.81, which indicated that correlation matrix was suitable for subsequent factor analysis. Using the Kaiser criterion, three factors with minimum eigenvalues >1.0 were identified that accounted for 58.6% of the total variance. Specifically, the three factors identified were interpreted as Factor 1 (shortness of breath, weakness, drowsiness and poor mobility), Factor 2 (poor appetite, insomnia, anxiety and depression) and Factor 3 (dry mouth and cough). The rotated loadings of the first three principal components are presented in Table 3.

**TABLE 3:** Factor loadings of exploratory factor analysis

Symptoms	Factor loadings		
	Factor 1	Factor 2	Factor 3
Shortness of breath	0.76	0.06	0.20
Weakness	0.69	0.41	0.00
Drowsiness	0.69	0.11	−0.09
Poor mobility	0.66	0.43	−0.01
Poor appetite	0.22	0.48	0.36
Insomnia	−0.11	0.55	0.50
Dry mouth	0.50	0.01	0.51
Cough	0.03	−0.05	0.79
Anxiety	0.25	0.79	−0.02
Depression	0.17	0.82	−0.02
Kaiser–Meyer–Olkin	0.81		
Bartlett sphericity Test	<0.05		

#### 4.2.2 Confirmatory factor analysis (CFA)

Following a three‐factor solution identified by EFA, we performed CFA to further examine the factor model. However, the results of CFA identified that the model emerging from EFA did not have a good fit (χ^2^/df = 3.48, *p *< .05; CFI = 0.92, TLI = 0.89; SRMR = 0.056, RMSEA = 0.074). Based on the cut‐off of 0.50 for factor loadings (Hair et al., 2014), we subsequently modified the factor model since it was uncertain whether poor appetite and insomnia should be incorporated into Factor 2 or Factor 3, and whether dry mouth should be incorporated into Factor 1 or Factor 3. No further modifications of the model structure were made since the final fit indices indicated an adequate model fit (χ^2^/df = 2.89, *p *< .05; CFI = 0.94, TLI = 0.92; SRMR = 0.050, RMSEA = 0.065). Consequently, we identified three symptom clusters: a respiratory related symptom cluster (shortness of breath, weakness, drowsiness and poor mobility), a psychological symptom cluster (anxiety and depression) and a cough‐insomnia related symptom cluster (poor appetite, insomnia, dry mouth and cough).

### 4.3 Structural equation modelling

#### 4.3.1 Hypothesised Model

**FIGURE 2:**
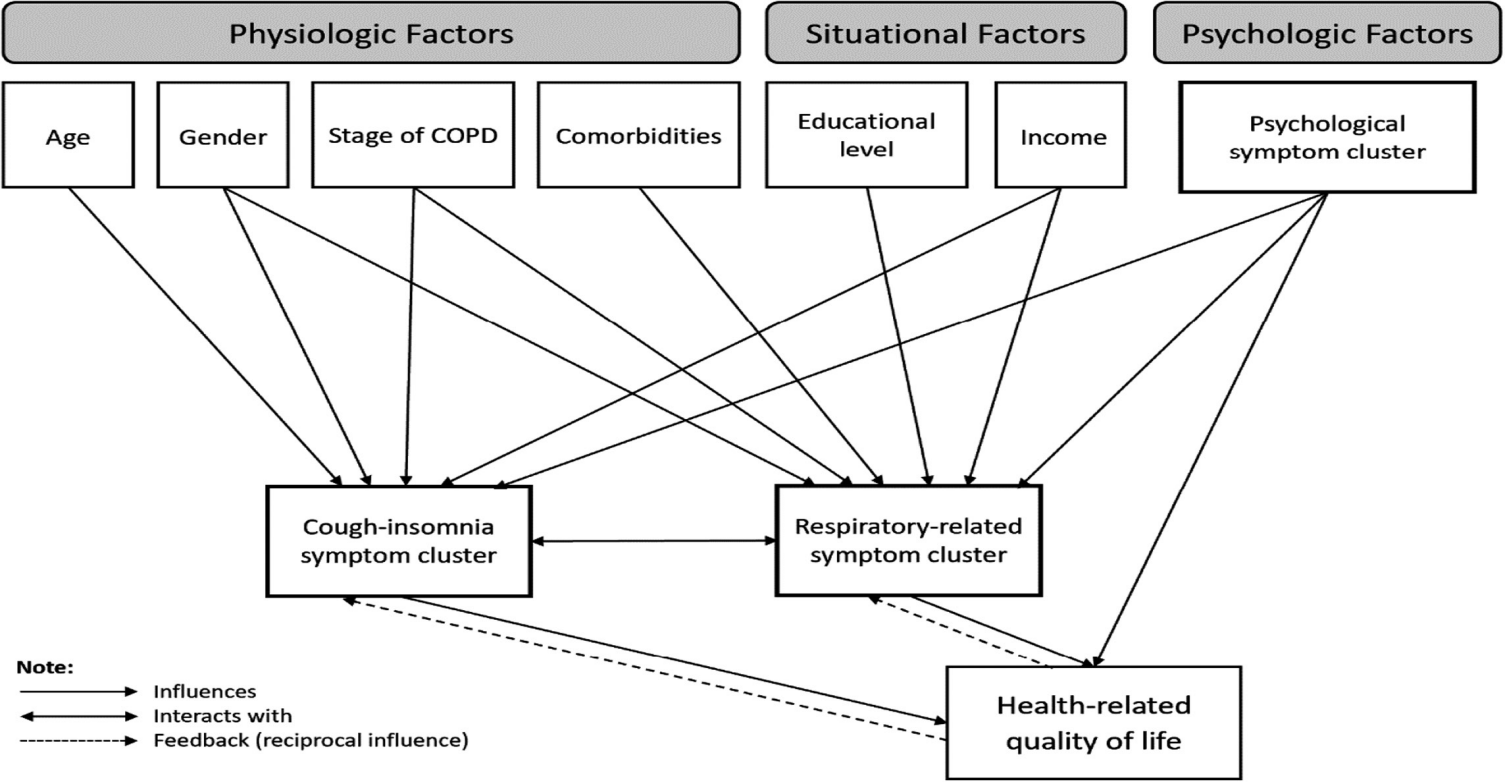
Hypothetical model

Based on the TOUS and literature review, we proposed a hypothesised model (refer to Figure 2). According to our systematic review (Fei et al., 2021), age, gender, educational level, income, comorbidities and stage of disease were consistently reported with individual symptoms in the research field of symptom. To construct the specific ‘path diagrams’, we performed a Kruskal–Wallis H test and Pearson's correlation analysis to examine the relationships between associated factors and symptom clusters. The detailed results are presented in Table 4. Although the results of Table 4 display some non‐significant relationships, age and monthly income were previously reported to be associated with the ‘fatigue‐insomnia cluster’ (Lim et al., 2017), additionally, comorbid conditions are possibly related to the respiratory related symptoms (Diez‐Manglano et al., 2014). Therefore, we assumed that age and monthly income were associated with the cough‐insomnia symptom cluster; and comorbidities were associated with the respiratory related symptom cluster.

**TABLE 4:** Relationships between associated factors and symptom clusters

Variable	Respiratory related symptom cluster		Cough‐insomnia related symptom cluster	
	H	*p*‐value	H	*p*‐value
Gender	4.39	.04*	4.20	.04*
Age in years	0.24	.62	0.69	.41
Educational level	5.72	.02*	1.40	.24
Monthly income per person	30.25	<.01*	1.74	.19
Number of comorbidities	2.37	.50	8.12	.06
Stage of COPD	110.82	<.01*	37.41	<.01*

Variable	Respiratory related symptom cluster		Cough‐insomnia related symptom cluster	
	Correlation value	*p*‐value	Correlation value	*p*‐value
Psychological factor	0.46	.00*	0.35	.00*

* *p*‐value <.05

The viewpoints of dealing with psychological symptoms have led to conceptual confusion. Specifically, some researchers consider depression or anxiety as associated factors (Tankumpuan et al., 2015), while some others consider these symptoms are part of the disease itself (Herr et al., 2015). In this study, we treated the psychological symptom cluster (anxiety and depression) as an associated factor, aiming to be consistent with the TOUS and to minimise confusion. Based on previous studies that identified the association between the emotional symptom clusters and quality of life (Wu et al., 2020; Yang et al., 2020), we assumed that the psychological symptom cluster (anxiety and depression) had a direct impact on HRQoL in COPD patients. We also wanted to explore the interaction within the symptom clusters as the TOUS illustrated single symptoms interacted with each other. Based on the TOUS, we drew an ‘influence’ path from symptom clusters to HRQoL and a ‘reciprocal influence’ path from HRQoL to symptom clusters. Finally, a hypothetical model of symptom clusters with their associated factors and effects on HRQoL in COPD patients was developed (Figure 2).

#### 4.3.2 Model testing

**FIGURE 3:**
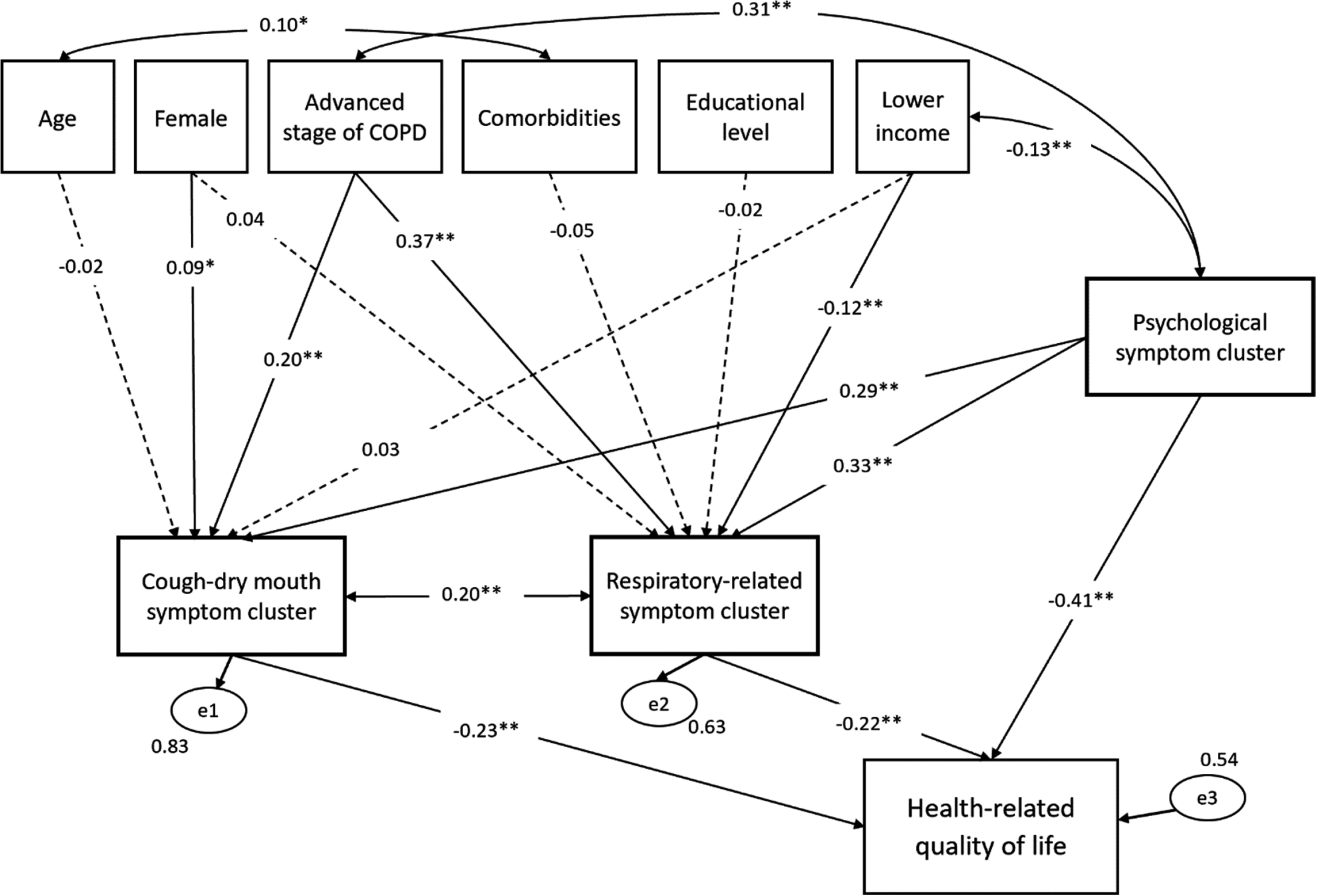
Final model with standardised coefficients. Note: The dashed lines with arrows represent non‐significant relationships (*p* > .05). *p < .05, **p < .01

Figure 3 illustrates the final model with standardised path estimates. Overall, we demonstrated that this model possesses good fit: χ^2^/df = 2.982, *p *< .05; CFI = 0.970, TLI = 0.920; RMSEA = 0.066, SRMR = 0.026. The reciprocal relationships between symptom clusters and health‐related quality of life were assessed several times; however, these relationships produced unstable estimates (taken hundreds of iterations). Therefore, the final model eliminates this proposed reciprocal relationship. The associated factors of respiratory related symptom cluster were the stage of COPD (*β* = 0.37, *p *< .0001) and income per month (*β* = −0.12, *p* = .004); the associated factors of cough‐insomnia related symptom cluster included being a female (*β* = 0.09, *p* = .048) in addition to the stage of COPD (*β* = 0.20, *p *< .0001). The psychological symptom cluster was significantly associated with the respiratory related symptom cluster (*β* = 0.33, *p *< .0001) and the cough‐insomnia related symptom cluster (*β* = 0.29, *p *< .0001); the respiratory related symptom cluster and the cough‐insomnia related symptom cluster interacted with each other (r = 0.20, *p *< .0001). Comorbidities did not have a significant effect on the respiratory related symptom cluster (*β* = −0.05, *p* = .24). Our findings indicate that the respiratory related symptom cluster, cough‐insomnia related symptom cluster and psychological symptom cluster all had a negative effect on HRQoL (*β* = −0.22, *p *< .0001; *β* = −0.23, *p *< .0001; *β* = −0.41, *p *< .0001, respectively). Together, the symptom clusters explained 46.33% of the variance in HRQoL.

## 5 DISCUSSION

To our knowledge, this is the first study to make use of a sophisticated statistical structural equation model to examine the complex relationship between symptom clusters, associated factors and resulting HRQoL in a large cohort of people living with COPD. Furthermore, making use of empirical data, the structural equation modelling enables the statistical testing of the phenomenon in addition to connecting this to theoretical concepts associated with symptom clusters (Bagozzi & Yi, 2012). Therefore, a major contribution of this study is that it provides both empirical and theory‐based evidence to inform clinical care.

In this study, we identified three robust symptom clusters (respiratory, psychological and cough/insomnia) among people living with COPD. In terms of the respiratory related symptom cluster, we identified that shortness of breath and weakness were the two most prevalent symptoms, at 89.5% and 80.4%, respectively. Comparisons of different scales used to assess symptoms, the particular timing of assessment, sample size and analytic techniques have also identified a ‘core’ symptom (breathlessness) constituting a respiratory related cluster (Lim et al., 2017; Wu et al., 2020; Yang et al., 2020). People with COPD who experience dyspnoea also frequently develop hypoxemia. Without adequate oxygen, many of these individuals will inevitably feel tired, fatigued and drowsy (Baltzan et al., 2011). Moreover, we identified poor mobility within the respiratory related symptom cluster, which was inconsistent with previous COPD studies (Lim et al., 2017; Yang et al., 2020) since they did not assess poor mobility. At the same time, we also identified a high prevalence (70%) of poor mobility, similar to a previous study (Shay et al., 2020). A cross‐sectional survey of 66,752 individuals indicated that older people with COPD might be more at risk of prolonged deconditioning associated with hospital stay and, as a consequence, experience mobility impairment (Fang et al., 2018). We also identified a cough‐insomnia related symptom cluster, previously observed among people with COPD in China (Wu et al., 2020). Specifically, Wu et al. (2020) identified a ‘GI’ symptom cluster (loss of appetite and dry mouth) and ‘disturbed sleep’ symptom cluster (cough and difficulty sleeping). Moreover, previous studies identified that cough and dry mouth were linked (Park & Larson, 2014; Yang et al., 2020); and people who suffered from difficulty sleeping were more likely to have a poor appetite (Ji et al., 2019). The four symptoms may be correlated with each other where the presence of cough acts as a ‘trigger’ that causes the development or exacerbation of the other three symptoms. Moreover, it is notable that the respiratory related symptom cluster and cough‐insomnia related symptom cluster interacted with each other. Whilst we know symptom clusters were relatively independent and the relationships among symptoms within a cluster were stronger than that among symptoms across different clusters (Kim et al., 2005), it seems reasonable that the symptom burden of these two clusters worsens synchronously over time since COPD is a progressive disease (Decramer & Janssens, 2013). In terms of the psychological symptom cluster, this finding is consistent with previous studies that reported individuals with COPD experienced concurrent anxiety and depression (Lim et al., 2017; Park & Larson, 2014).

Our final COPD symptom cluster model also depicts the key and specific physiological or situational contributors for each symptom cluster. This model identified those patient participants with a severe stage of COPD and with lower income were more likely to experience shortness of breath, weakness, drowsiness and poor mobility symptoms. This is in line with previous research (Lim et al., 2017). It is possible that people living in poor economic conditions and without access to private health insurance (Zhu et al., 2018) might not be able to benefit from treatments to mitigate their distress. Our findings also showed that among study participants with COPD, those who were female with advanced stage of disease were more likely to experience poor appetite, insomnia, cough and dry mouth. One possible explanation for this may be that being female was a significant independent determinant for insomnia in COPD patients (Winkelman et al., 2020). However, in our study, comorbidities were not significantly associated with any particular symptom cluster. Epidemiological studies have observed that COPD comorbidities included psychological disturbances, cardiovascular disease, skeletal muscle dysfunction, and other physical diseases (Clini et al., 2014). Moreover, COPD is a heterogeneous disease with multiple systemic characteristics and was reported to be based on the presence of common risk factors with other coexisting diseases (Franssen & Rochester, 2014). Therefore, the absence of a significant result in our study may be due to the overlap between comorbidities and other factors impacting symptom clusters.

In the final model, our study findings identify significant relationships between the symptom clusters and HRQoL among COPD patients. Our findings showed that the respiratory related symptom cluster and the cough‐insomnia related symptom cluster had a greater negative impact on HRQoL. Previous studies have consistently reported that respiratory related symptom cluster had negative effects on HRQoL in COPD patients (Park & Larson, 2014; Wu et al., 2020). Interestingly, if we treated the psychological symptom cluster as an associated factor aiming to maximise consistency with the TOUS, it should not have directly influenced HRQoL according to TOUS. However, the final model revealed that the psychological symptom cluster directly influenced HRQoL. If we treat the psychological symptom cluster as symptom experience, according to the definition of ‘symptom cluster’ (Kim et al., 2005), the psychological symptom cluster should be independent of the other two symptom clusters. Contrary to this definition, the direct path from the psychological symptom cluster to the other two symptom clusters in the final model was significant, suggesting the psychological symptom cluster was not independent of the other two symptom clusters. Based on these results, we summarised that whatever the role of psychological symptom cluster was, it could directly influence HRQoL or through symptom clusters indirectly influence HRQoL in COPD patients, which formed a closed circle between the psychological symptom cluster, respiratory related symptom cluster (or cough‐insomnia related symptom cluster) and HRQoL. It has been postulated that people who experience depression and anxiety disorder may be more susceptible to COPD because they have a greater risk of smoking (Hanania et al., 2011) and that depression and anxiety may be associated with a subsequent inflammatory response to this disease (Van Dijk et al., 2004). Although the aetiology of anxiety and depression among people with COPD is not fully understood, the correlation between COPD and anxiety and depression might lead to a ‘self‐perpetuating’ situation that has a negative influence upon a patient's HRQoL (Pumar et al., 2014). As far as we know, this research study is the first to identify and quantify the strength and direction of these relationships among COPD patients. Our findings reinforce the importance of focusing on screening for anxiety and depression with potential successive treatment could be promising approaches to reduce symptom burden and improve HRQoL in COPD patients.

In the final model, we were not able to support a response loop from HRQoL to symptom clusters as suggested by the TOUS. Conceptually, the outcomes, for example, HRQoL influences the experience of concurrent symptoms seems to be reasonable (Lenz et al., 1997). The data analysed in this research are cross‐sectional, and consequently, it may be not possible to determine the order of the loop (Martens & Haase, 2006). Future COPD symptom cluster‐based research should focus on a longitudinal time‐lagged design, possibly leading to more stable estimates.

### 5.1 Limitations

This study has several limitations. First, this study was conducted in a Chinese context, therefore, the generalisability of this study’s findings will not be assured elsewhere. Second, the cross‐sectional design provides a relatively lower level of evidence. However, we adopted a powerful method analysis namely structural equation modelling to mitigate in part this concern. In future, longitudinal research is needed to determine the causal relationships and test the reciprocal relationships among the study variables. Third, the questionnaires employed in this research may be affected by factors such as memory bias, social desirability and reporting bias (Lau et al., 2017). Finally, potential biological factors, such as the inflammatory biomarker C‐reactive protein (CRP) and procalcitonin (PCT; Fei et al., 2021), were not captured in this study. This warrants future research.

## 6 CONCLUSIONS

In this study, we have quantified for the first time the relationship between symptom clusters and associated factors and HRQoL in people living with COPD. Identifying symptom clusters and their associated factors is of great significance for nurses in the identification of symptom synergism and further guaranteeing effective symptom assessment and management. Additionally, our data serves as a platform for future research to develop cluster‐targeted assessment and interventions, to guide nursing care to reduce the symptom burden of patients living with COPD and enhance their quality of life.

## 7 RELEVANCE TO CLINICAL PRACTICE

Our findings have practical implications for nurses involved in COPD care. Specifically, nurses should be more mindful to identify patients who may be at risk of facing a higher symptom burden during nursing admission assessment, in this case, those who are female, those who have advanced stage of COPD and those with lower incomes. Moreover, if nurses are empowered with knowledge of symptoms that typically co‐occur, they can then pay more attention to the nature of the correlations among symptoms within a cluster rather than focusing on the symptom itself. This could potentially help nurses to uncover the ‘trigger’ symptom that could cause the development or exacerbation of other symptoms. It is plausible that nurses may conduct a management strategy targeted at the ‘trigger’ symptom in a cluster, then other symptoms in the same cluster may be relieved correspondingly. Furthermore, nurses need to conduct a routine assessment and early detection of anxiety and depression, since these symptoms were responsible for a profound negative effect on HRQoL and had also indirect effects on HRQoL by influencing other symptom clusters. Interventions to promote self‐management behaviours that address anxiety and depression should be ongoing and tailored for COPD patients that may have the potential in improving HRQoL. We believe this symptom cluster model we present may serve as a framework to guide appropriate intervention research to improve COPD patients’ HRQoL. Future research is also needed to further examine the diversified interactions between concurrent symptoms on biology, psychology or sociology, which may contribute to detecting the ‘trigger’ symptom or the underlying aetiology shared by symptoms within a cluster, and then could develop effective symptom management strategies based on the identification of core symptoms and symptom clusters using the novel approach of Network Analysis (NA).

## CONFLICT OF INTEREST

No conflict of interest has been declared by the authors.

## AUTHOR CONTRIBUTIONS

The study and data collection tools were designed by and FF, JK and WG. FF and XZ were responsible for patient recruitment and data collection. JK, RS and WG guided FF and XZ with the data analysis. All authors reviewed the findings, agreed with the interpretation, contributed to writing the paper, had full access to all data in the study, and read and approved the final version.

## Supporting information

Supplementary Material

## Data Availability

The data that support the findings of this study are available on request from the corresponding author. The data are not publicly available due to privacy or ethical restrictions.
